# Round Spermatid Injection Rescues Female Lethality of a Paternally Inherited *Xist* Deletion in Mouse

**DOI:** 10.1371/journal.pgen.1006358

**Published:** 2016-10-07

**Authors:** Federica Federici, Aristea Magaraki, Evelyne Wassenaar, Catherina J. H. van Veen-Buurman, Christine van de Werken, Esther B Baart, Joop S. E. Laven, J Anton Grootegoed, Joost Gribnau, Willy M Baarends

**Affiliations:** 1 Department of Developmental Biology, Erasmus MC, University Medical Center, Rotterdam, The Netherlands; 2 Division of Reproductive Medicine, Department of Obstetrics and Gynaecology, Erasmus MC, University Medical Center, Rotterdam, The Netherlands; Friedrich Miescher Institute for Biomedical Research & University of Basel, SWITZERLAND

## Abstract

In mouse female preimplantation embryos, the paternal X chromosome (Xp) is silenced by imprinted X chromosome inactivation (iXCI). This requires production of the noncoding *Xist* RNA in *cis*, from the Xp. The *Xist* locus on the maternally inherited X chromosome (Xm) is refractory to activation due to the presence of an imprint. Paternal inheritance of an *Xist* deletion (Xp*ΔXist*) is embryonic lethal to female embryos, due to iXCI abolishment. Here, we circumvented the histone-to-protamine and protamine-to-histone transitions of the paternal genome, by fertilization of oocytes via injection of round spermatids (ROSI). This did not affect initiation of XCI in wild type female embryos. Surprisingly, ROSI using *ΔXist* round spermatids allowed survival of female embryos. This was accompanied by activation of the intact maternal *Xist* gene, initiated with delayed kinetics, around the morula stage, resulting in Xm silencing. Maternal *Xist* gene activation was not observed in ROSI-derived males. In addition, no *Xist* expression was detected in male and female morulas that developed from oocytes fertilized with mature *ΔXist* sperm. Finally, the expression of the X-encoded XCI-activator RNF12 was enhanced in both male (wild type) and female (wild type as well as Xp*ΔXist*) ROSI derived embryos, compared to *in vivo* fertilized embryos. Thus, high RNF12 levels may contribute to the specific activation of maternal *Xist* in Xp*ΔXist* female ROSI embryos, but upregulation of additional Xp derived factors and/or the specific epigenetic constitution of the round spermatid-derived Xp are expected to be more critical. These results illustrate the profound impact of a dysregulated paternal epigenome on embryo development, and we propose that mouse ROSI can be used as a model to study the effects of intergenerational inheritance of epigenetic marks.

## Author Summary

In sexual reproduction, maternal and paternal haploid sets of DNA are combined in one new diploid individual. However, not only DNA, but also epigenetic information, defined by DNA and histone modifications, is transferred to the zygote. Specific inactivation of the paternally inherited X chromosome (Xp) in the preimplantation female mouse embryo is required for embryo survival. This imprinted X chromosome inactivation (iXCI) is initiated by transcription of the *Xist* gene from Xp. In contrast, the maternal *Xist* gene is imprinted during oogenesis to remain silent. We have investigated the consequences of elimination of the histone-to-protamine and protamine-to-histone transitions on iXCI, by fertilization through injection of immature round spermatids into oocytes (ROSI). Interestingly, when the round spermatids used for ROSI carried an X chromosome with an *Xist* deletion (*ΔXist*), we found that the *Xist* gene on the maternal X chromosome was activated, which rescued the female lethality of embryos that is invariably observed upon fertilization with mature *ΔXist* spermatozoa. This striking result is best explained by deregulation of embryonic gene expression, in particular from Xp, when the paternal genome originates from round spermatids rather than spermatozoa. From this, we suggest that the use of round spermatids has unforeseen consequences for embryonic gene expression and its use in human assisted reproduction must be carefully considered.

## Introduction

In mammals, as in all diploid organisms with a sexual reproduction cycle, the diploid zygote is formed upon fertilization by combination of the haploid maternal and paternal genomes. Sperm and egg each contribute a complete set of chromosomes, and in addition the gametes carry sex-specific epigenetic information that is important for correct execution of the early developmental gene expression program. A striking epigenetic difference between the paternal and maternal epigenomes is caused by the fact that the paternal chromatin undergoes two rounds of complete remodelling in the reproductive cycle. First, during the final post-meiotic phase of spermatogenesis, in elongating and condensing spermatids, the vast majority of histones is replaced by protamines, generating the compact sperm nucleus. Second, immediately following fertilization, the protamines are replaced by maternal histones. The maternally provided histones H3 and H4 on the paternal pronucleus are devoid of lysine di- and tri-methylation marks, which leads to clear global differences in heterochromatin organization between the paternal and maternal genomes that are maintained up to the 8-cell stage [1,2]. In addition to a haploid set of autosomes, a spermatozoon contributes either an X or a Y chromosome to the zygote. The sex chromosomes are more drastically remodelled than the autosomes during spermatogenesis, because the heterologous X and Y chromosomes undergo meiotic sex chromosome inactivation (MSCI) in spermatocytes (reviewed by [3]), which is associated with chromosome wide nucleosome exchange [4]. After meiosis, silencing of X- and Y-linked genes is largely maintained during spermatid differentiation through post-meiotic sex chromatin repression (PSCR) [5]. Noteworthy, a number of X- and Y-linked genes, single and multi-copy, escape PSCR and become specifically reactivated [5–7] until the global transcriptional silencing that accompanies the histone-to-protamine transition sets in, in condensing spermatids. Subsequently, after fertilization, the X chromosome of paternal origin (Xp) will always be inactivated in female pre-implantation embryos and this is maintained in the extra-embryonic tissues of post-implantation embryos. This imprinted X chromosome inactivation (iXCI) depends on expression and spreading *in cis* of the *Xist* noncoding RNA on the Xp [8]. X-encoded RNF12 is a known and important XCI *trans* activator, acting through a dose-dependent mechanism in the activation of *Xist* transcription [9–11]. Maternal expression of RNF12 has been shown to be essential for iXCI, whereas deletion of the paternal copy is compatible with normal female embryo development and establishment of iXC [9]. Thus, the inactive state established on the Xp by MSCI and PSCR in spermatogenesis is not directly transmitted to female pre-implantation embryos but has to be re-established. However, whether the epigenetic events associated with the presence of unsynapsed chromatin are involved in establishing a paternal imprint at the Xic is not fully clear. In one study, expression of the *Xist* transgene was observed in preimplantation embryos only when the transgene was inherited from the father, independently of hemi- or homozygosity, indicating that imprinting was normally established on the single copy *Xist* transgene[12]. However, in a more recent study, correct imprinted expression was observed only when transgenic inserted *Xist* was transmitted from a hemizygous father[13]. Here the transgene was present in multiple copies. Irrespective of the mechanistic background of the paternal Xic imprint, it is clear that paternal X-linked genes are transcriptionally active at the 2-cell stage and are then gradually inactivated *de novo* via *Xist* RNA-dependent silencing [12,14]. During iXCI *Xist* RNA spreading on the Xp triggers the recruitment of chromatin-modifying protein complexes, which in turn will establish repressive epigenetic marks on the Xp, rendering it transcriptionally inactive. At the blastocyst stage, while iXCI is stably maintained in extra-embryonic tissues, the Xp becomes reactivated in the epiblast followed by random XCI without a parent-of-origin bias [15]. Paternal inheritance of a *Xist* deletion *(ΔXist*) completely abolishes iXCI of the Xp [16], arrests development around E6.5, and results in reabsorption of mutant female embryos by E12.5 [17]. The strong bias towards Xp inactivation in iXCI is favoured by the presence of an imprinting mark on the X chromosome of maternal origin (Xm) that prevents it from expressing *Xist* [18,19]. In addition, Xp may also be imprinted to become preferentially inactivated, as might be inferred from the recently reported effects from the pairing status of an *Xist* transgene during male meiotic prophase [13], although the nature of such an imprint remains elusive. It has been proposed that iXCI occurs as a two-step process [20]. First, pre-inactivated intergenic repeat regions on the Xp may carry transgenerational epigenetic information from the paternal germline to the zygote, predisposing the Xp for iXCI independently of *Xist* [20]. This might rely on the inheritance of sperm-derived nucleosomes and their associated modifications. Second, subsequent establishment of genic silencing strictly depends on *Xist* expression from the paternal allele [20]. Alternatively, it has been suggested that the preferential inactivation of Xp may simply rely on early and robust activation of the paternal *Xist* gene [21]. This may be facilitated, upon fertilization, by the protamine-to-histone transition, during which the protamine-based chromatin of the sperm acquires newly deposited histones lacking most heterochromatic marks. The transcriptionally permissive chromatin signature deposited on the haploid genome in the paternal pronucleus would then allow *Xist* expression from the paternal allele. Conclusive evidence for the contribution of the protamine-to-histone transition in the initiation of iXCI is lacking.

To test if the chromatin rearrangement in spermatids impacts on iXCI, we made use of mouse round spermatids to fertilize oocytes. When a round spermatid is injected into a mouse oocyte (ROSI), the paternal genome has a histone-based chromatin constitution, contrary to the protamine-packaged chromatin of spermatozoa. Hence, ROSI evades the protamine-to-histone replacement in the male pronucleus, but rather provides for a paternal genome with a spermatogenic histone-based chromatin composition. Here, by using ROSI as an experimental tool, and a method to visualize individual chromosomes in fixed early embryos, we could establish that the chromatin constitution of the X chromosome in round spermatids is maintained in ROSI-derived zygotes. Next, we observed that absence of genome wide paternal chromatin remodelling did not affect the timing of *Xist* expression in ROSI-derived female zygotes, on a wild type background. We then asked if the transcriptionally repressed state and the heterochromatin marks present on the X chromosome of round spermatids, because of MSCI and PSCR, might be sufficient to establish iXCI independently of *Xist*-mediated silencing. This was tested using round spermatids from male mice lacking a functional *Xist* gene (Xp*ΔXist*) for ROSI. Here, we would expect rescue of the early embryonic lethality of Xp*ΔXist* female embryos through maintenance of MSCI- and PSCR-mediated inactivation of Xp, which is *Xist*-independent. Our results showed that injection of Xp*ΔXist* round spermatids indeed prevents the female lethality, that is always observed upon fertilization with mature Xp*ΔXist* spermatozoa. Surprisingly, this rescue occurred through inactivation of the Xm and not by *Xist*-independent Xp silencing. In addition, we observed high levels of the XCI activator RNF12, in all ROSI derived embryos, independent of sex and genotype. These findings are discussed in the context of our current views on iXCI, and possible implications for transfer of dysregulated paternal epigenetic information to the embryo are described in relation to the clinical application of assisted reproductive technology.

## Results and Discussion

### Histones and associated epigenetic modifications are transmitted from round spermatids to ROSI-derived zygotes

To analyze the histone modification patterns of round spermatid-derived paternal chromatin in early embryos, and in particular the epigenetic profile of the Xp, we arrested ROSI-derived mouse embryos at the pro-metaphase stage of the first or second cleavage divisions [22]. This allows the visualization of epigenetic marks on individual chromosomes. As a control for staining specificity, zygotes obtained by intracytoplasmic sperm injection (ICSI), using epididymal spermatozoa, were subjected to the same experimental procedure.

**Fig 1:**
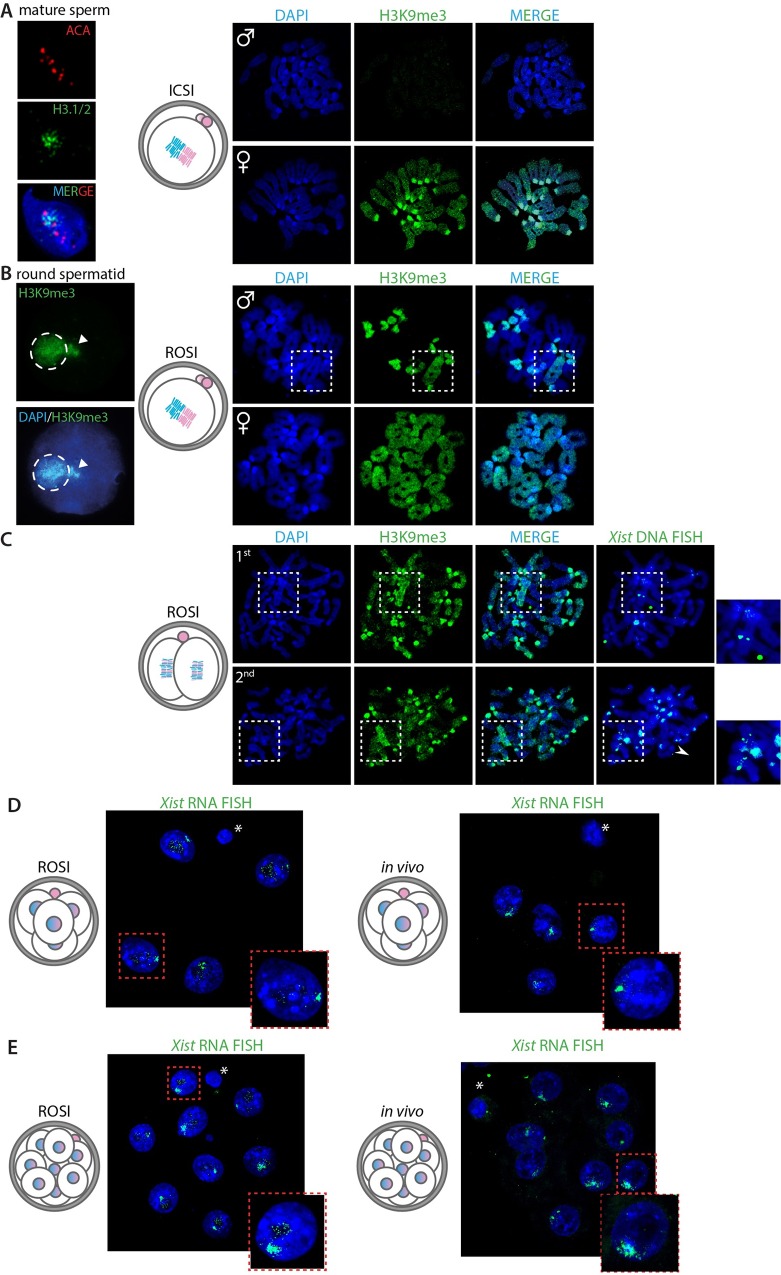
Chromatin remodeling and *Xist* expression in ROSI-derived embryos. A) Left panel: Representative image of a decondensed nucleus of a mature mouse spermatozoon stained with H3.1/2 (green) and anti-centromere antibody (ACA) (red), to illustrate the limited presence of histones in association with pericentromeric chromatin in mature mouse sperm, as shown previously by others [24]. Right panels: Representative image of immunolocalization of H3K9me3 (green) in chromosome spreads of prometaphase-arrested mouse zygotes obtained by ICSI (as represented by the drawing, n = 3 zygotes). The condensed chromosomes of paternal and maternal origin are cutouts from the whole zygote images (paternal chromosome set on top; maternal below). DNA is counterstained with DAPI (blue). B) Left panel: Representative image of a nucleus of a mouse round spermatid immunostained for H3K9me3 (green), to illustrate the enrichment of H3K9me3 on the chomocenter (encircled) and adjacent sex chromosome (arrowhead) as described previously [4]. Right panels: Representative image of immunolocalization of H3K9me3 (green) in chromosome spreads of prometaphase-arrested zygotes obtained by ROSI (as represented by the drawing, n = 20 zygotes). The condensed chromosomes of paternal and maternal origin are cutouts from the whole zygote images (paternal chromosome set on top; maternal below). The X chromosome is indicated by a white dashed square box, whose identity is inferred from the known enrichment of the round spermatid X chromosome for this marker [5]. C) Representative image of immunofluorescence analysis for H3K9me3 (green) on chromosome spreads of a representative prometaphase-arrested 2-cell stage embryo obtained by ROSI (as indicated by the drawing on the left, n = 2). Each blastomere from the same embryo is cutout into separate images (1^st^ blastomere on top; 2^nd^ blastomere below). The X chromosome is indicated by a white dashed square box. *Xist* DNA FISH (left panel) was performed on the same chromosome spreads represented on the left. Square boxes on the right are blowups of each corresponding boxed area containing one X chromosome (1^st^ blastomere, one X is not visible) or two X chromosomes (2^nd^ blastomere). D) Representative images of *Xist* RNA FISH on a ROSI-derived 4-cell stage embryo on the left (n = 5 ROSI embryos), and a 4-cell stage embryo derived by *in vivo* fertilization (n = 4). Dashed red square boxes are blowups of each corresponding boxed area. E) Representative image of *Xist* RNA FISH on a ROSI-derived 8-cell stage embryo on the left (n = 5 ROSI embryos), and an 8-cell stage embryo derived by *in vivo* fertilization. Dashed red square boxes are blowups of each corresponding boxed area.

Following the histone-to-protamine transition in spermatids, mouse epididymal spermatozoa contain approximately 1% of residual histones [23,24]. After sperm decondensation by heparin treatment and immunostaining for histone H3.1/2 and centromeres (with anti-centromere antibody, ACA), limited histone retention associated with pericentromeric regions was visible (Fig 1A, left panel), as previously shown [24]. After fertilization, when the protamine-to-histone transition has taken place, we did not detect any H3K9me3 at paternal prometaphase chromosomes of ICSI-derived zygotes (Fig 1A, right panel), while prometaphase chromosomes of maternal origin were strongly enriched for H3K9me3 at pericentromeric regions and displayed moderate H3K9me3 levels along the chromosome arms. These results are in accordance with results from previous studies on *in vivo* fertilized embryos, that showed epigenetic asymmetry between maternally and paternally inherited chromatin up to the third cleavage division [1,2,25,26].

In round spermatids, the X and Y chromosomes, as well as the constitutive pericentric heterochromatin clustered in the chromocenter, are enriched for H3K9me3 (Fig 1B, left panel), in accordance with previously published data [4]. In ROSI-derived zygotes at the first cleavage division, we detected persistence of H3K9me3 at the DAPI-dense heterochromatic chromosome ends of paternal origin and on the entire Xp (Fig 1B, right panel). This epigenetic profile mirrors the H3K9me3 pattern observed in round spermatids. ROSI-derived 2-cell stage female embryos that were arrested at the pro-metaphase of the second cleavage division displayed maintenance of a high enrichment for H3K9me3 in particular on one of the two X chromosomes (Fig 1C), as confirmed by *Xist* DNA FISH (note that some residual H3K9me3 signal generates background staining after the FISH procedure Fig 1C, right panel and enlargements). One blastomere (the lower one, in Fig 1C) showed DNA FISH staining also on the Xm, confirming that the embryo was indeed female.

The present results are in agreement with previous observations, that a substantial fraction of modified histones present in round spermatid chromatin is maintained in early pre-implantation embryos generated by ROSI [27]. Here, we show that this concerns in particular the X chromosome, where the H3K9me3 chromatin signature covers the entire chromosome.

### Normal establishment of iXCI in ROSI-derived female zygotes

We then aimed to investigate if absent or limited remodeling of paternal chromatin in ROSI-derived female zygotes, and the heterochromatic epigenetic signature of the X chromosome specifically, might affect the timing of *Xist* expression and interfere with iXCI establishment. In ROSI-derived female embryos, *Xist* expression started normally at the 4-cell stage (Fig 1D), when clear *Xist* RNA FISH clouds are already visible, and these are further enhanced at the 8-cell stage (Fig 1E), similarly to what has been described for *in vivo* fertilized control embryos [28]. We cannot discriminate between the two parental X chromosomes in this experiment, but since we never observed *Xist* clouds,nor *Xist* mRNA expression above background in male wild type ROSI-derived morulas (see below), and since the timing of *Xist* expression is normal, we infer that it is the Xp that is inactivated. Thus, the data suggest that the protamine-to-histone transition does not play a major role in the activation of the paternal *Xist* gene. In addition, the enrichment of H3K9me3 along the X chromosome does not appear to interfere with *Xist* transcription. It therefore seems more likely that another type of imprint, or a different mechanism, controls preferential *Xist* expression from Xp.

### Transmission of Xp*ΔXist* through round spermatids rescues female embryonic lethality

Female embryos inheriting an *Xist* deletion on the Xp can no longer be recovered by E12, because lack of imprinted inactivation of the Xp leads to embryonic lethality [16]. We tested if transmission of an Xp carrying the *Xist* deletion through ROSI, instead of fertilization with mature sperm, might rescue the embryonic lethal phenotype. This experiment was based on the hypothesis that inheritance of an Xp that carries the transcriptionally silenced PSCR heterochromatic signature, might provide sufficient dosage compensation of X-linked genes in the absence of *Xist*, and thereby would allow female embryo survival. We performed ROSI with XpΔXist round spermatids from the C57BL/6 *ΔXist* mouse line generated by Csankovszki et al. [29]. We also performed ICSI with Xp*ΔXist* mature sperm from the C57BL/6 mouse line. At E15, we obtained 17 pups after ROSI and 11 after ICSI (Table 1). This was approximately 10% of the number of 2-cell stage embryos that were transferred to pseudopregnant females, and 25% of the counted implantations, for both techniques. The sex of the embryos was determined by visual inspection of the isolated gonads and confirmed by genotyping PCR for all females and most males (S1A and S1B Fig). The ICSI experiments yielded only males, as expected. In contrast, 5 out of the 17 E15 embryos generated by ROSI were female (p<0.05, chi square test). All male and female surviving embryos appeared normal in size and appearance and there was no overt difference between ICSI versus ROSI embryos, or between males and females. Interestingly, female embryos could not be generated when using Xp*ΔXist* spermatids from *M*. *musculus castaneus* (CAST/EiJ) males (Table 1, male sex assessed by the presence of testes). Male fertility parameters of CAST/EiJ mice differ significantly from those of C57BL/6 mice [30]. Although CAST/EiJ males are normally fertile, this result indicates that there may be critical differences in gene expression between the two subspecies. At present we cannot point to any specific causal difference that would explain our failure to rescue on this background. For all our subsequent experiments we continued with C57BL/6 spermatids.

**Table 1:** 

	ICSI C57BL/6 (E15)	ROSI C57BL/6 (E15)	ROSI C57BL/6 (P0)	ROSI CAST/EiJ (E15)
**#** **of independent experiments**	4	4	2	3
**#** **of 2-cell embryos transferred**	104	163	64	156
**#** **of pups**	11	17	7	12
**#** **of XY Males**	11 (2,3,1,5)	12 (1,5,1,5)	5 (2,3)	12 (2,5,5)
**#** **of XX Females**	0	**4 (0,1,1,2)**	**2 (0,2)**	0
**#** **of XO Females**	0	1(1,0,0,0)	0	0

#; number, numbers in brackets indicates number of animals in each experiment

**Fig 2:**
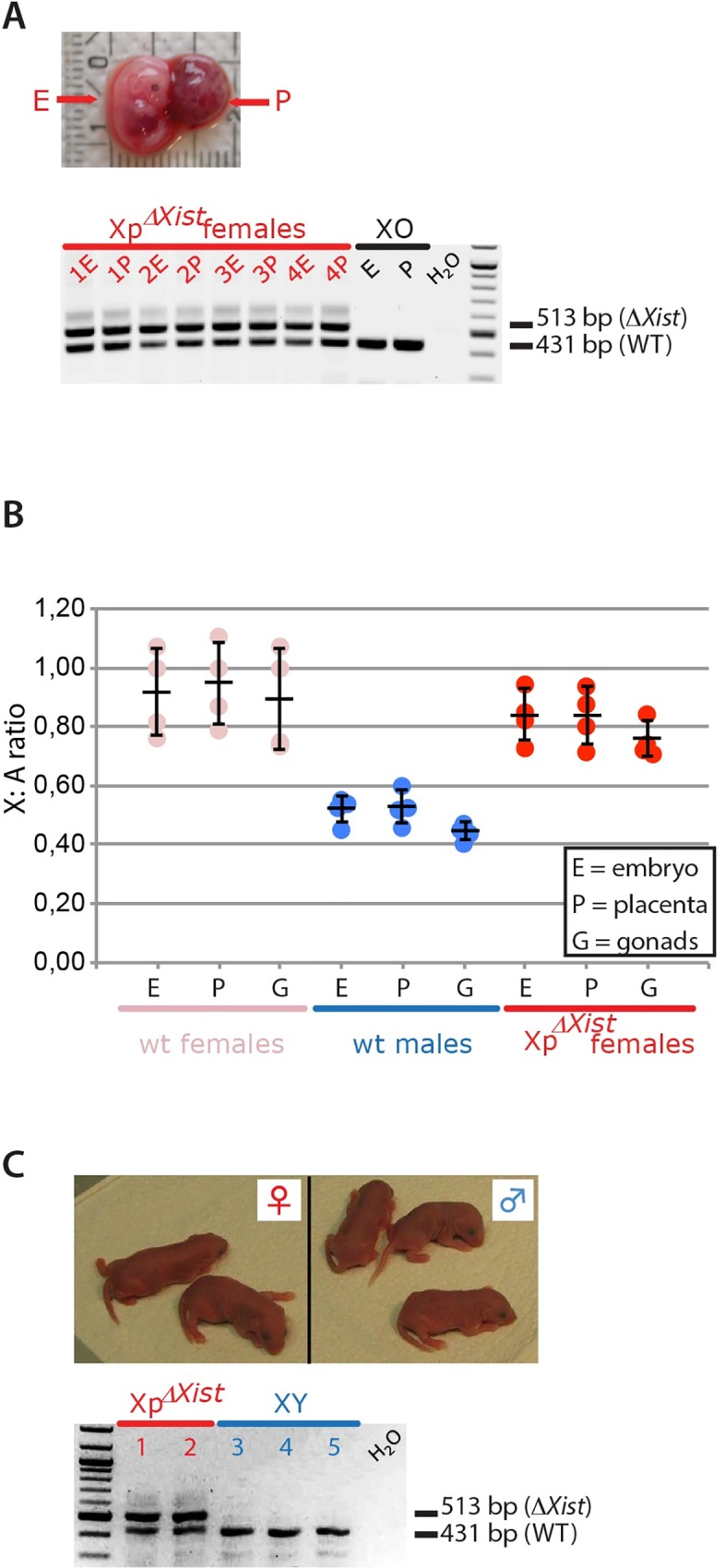
ROSI rescues female-specific lethality of a paternally inherited *Xist* deletion (Xp*ΔXist*). A) Representative E15 Xp*ΔXist* ROSI-derived female embryo (E) and attached placenta (P). Genotypes on DNA isolated from embryo and placenta of 4 ROSI-derived Xp*ΔXist* E15 female embryos and the single ROSI-derived XO embryo are shown below. Genotype was determined by the presence of a PCR product for the wt *Xist* allele (431bp band) and deleted allele (513 bp band). Water negative control and 100 bp marker were also loaded. B) Bar graph showing X:A ratios determined for different tissues in E15 embryos as indicated below the X-axis. To determine the X:A ratios, qPCR on genomic DNA isolated from E15 embryos, placentas and gonads was performed for *Xist* (chromosome X) and *Rex1* (chromosome 8). Results for each gene in each tissue were normalized to the values obtained in one reference wild type female. The X:A ratio was then determined for each individual tissue in 4 wild type females, 4 wild type males and in the 4 Xp*ΔXist* ROSI-derived female embryos. Results for each individual tissue are shown as dots, the average values (black horizontal line) and standard deviations (error bars) are also indicated. C) Image of 5 newborn pups with normal appearance derived with ROSI using Xp*ΔXist* round spermatids (2 females on the left and 3 males on the right). Genotypes for the wt and deleted *Xist* alleles are shown below. As expected, both females were heterozygotes, while the males only had the wt *Xist* allele inherited from the mother.

We genotyped all ROSI-derived female embryos, and found both a wild type *Xist* allele and a deleted *Xist* allele in embryonic and extraembryonic tissues of four of the female embryos, while one embryo was an XO female which had lost the mutated paternal X chromosome (Fig 2A). Next, we analysed the X chromosome to autosome ratio (X:A) for all embryos using quantitative PCR on genomic DNA, and observed the expected 1:1 ratio for the four XX embryos, and their placentas and isolated gonads (Fig 2B).

We then verified if ROSI-derived Xp*ΔXist* female embryos might develop to term. To this end, we collected pups in the morning after birth (P0). We obtained 7 live born pups (10% survival of 2-cell embryos that were transferred, Table 1), of which 5 were males and 2 were females. Sex was confirmed by PCR for *UbeX* and *UbeY* (S1C Fig). The two Xp*ΔXist* females were comparable in size and body weight to the male wild type siblings (Fig 2C). Genotyping for the mutated and wild type *Xist* allele confirmed heterozygosity of these females (Fig 2C). Our recovery of embryos following ICSI or ROSI is relatively low, compared to previously published data [31]. Part of the low recovery yield from the ICSI can be explained by the lethality of females due to the paternal *Xist* deletion. For the ROSI experiments, the rescue that we observe appears to be only partial. This is based on a comparison between the male to female sex ratio of 2.83 observed when Xp*ΔXist* ROSI embryos and pups are taken together (n = 23), and published sex ratios observed in 12 different published mouse ROSI experiments, involving comparable numbers of mice per experiment, whereby a mean male:female sex ratio of 0.93 ±0.48 was observed, with a maximum observed sex ratio of 2.33 (S1 Table).

### ROSI with Xp*ΔXist* allows initiation of XCI on the maternal X chromosome

**Fig 3:**
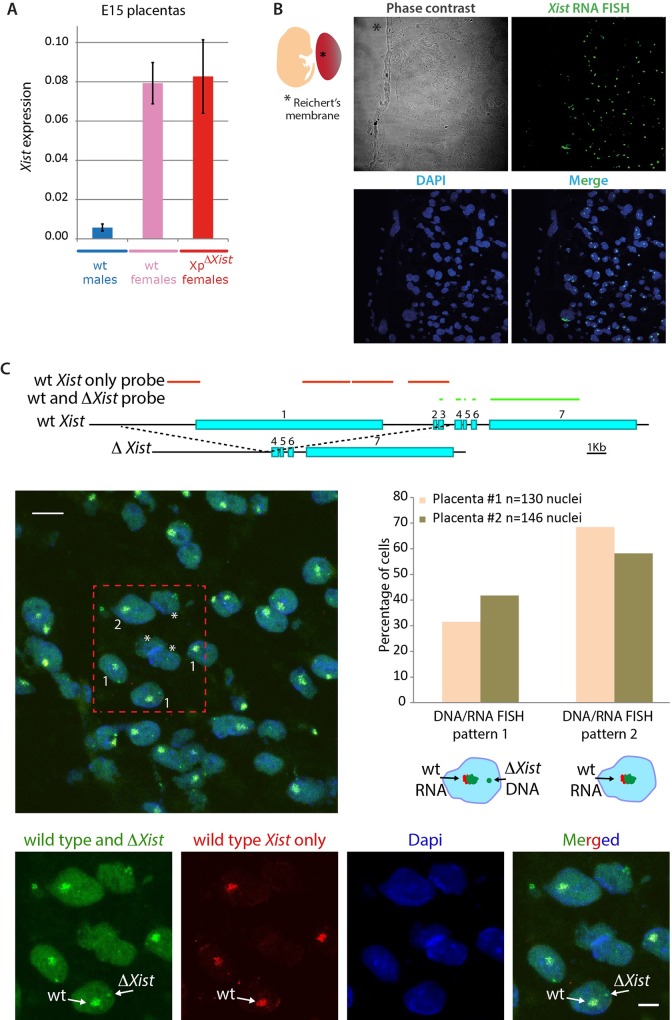
*ΔXist* female survival is mediated by a shift to inactivation of the maternal X (Xm). A) Average *Xist* gene expression levels ± s.d. on RNA isolated from E15 placentas of 3 control males (light blue bars), 3 control females (pink bars) and 3 ROSI-derived XpΔXist females (red bars). The data were normalized to actin. B) Representative image of the *Xist* RNA FISH (in green) on cryosections from a E15 ROSI-derived Xp*ΔXist* female placenta (n = 2). From the phase contrast image on top (right), the Reichert’s membrane on the embryonic side of the placenta can be visualized (marked by asterisk). DNA is counterstained with DAPI. C) Top: Schematic drawing of the RNA/DNA FISH probes used to detect the wild type (wt) *Xist* and the *ΔXist* gene and/or RNA. Exon numbers are indicated. The green probe localizes to sequences present in both the wild type *Xist* and *ΔXist* gene, whereas the red probe localizes to sequences that are only present in the wild type *Xist* gene (for further details see the Materials and Methods section). Left: representative merged image of the *Xist* RNA/DNA FISH using a probe recognizing the RNA produced by the wild type maternal allele and the DNA of both wild type *Xist* (signal is hidden under *Xist* RNA FISH cloud) and *ΔXist* alleles (green), and a probe recognizing only the DNA of the wild type *Xist* allele (hidden under *Xist* RNA FISH cloud) and RNA produced by the wild type maternal X chromosome (red) on cryosections from a E15 ROSI-derived Xp*ΔXist* female placenta (n = 2). DNA (Dapi) is shown in blue. Examples of nuclei are shown with an RNA FISH cloud signal with both probes, and a separate DNA FISH pinpoint in green (type 1), or with only an RNA FISH cloud signal with both probes (type 2). Cells lacking a cloud (some nuclear sections may not include the Xi) or with complicated staining patterns (due to the presence of polyploid cells, as was documented previously [53]) were not counted. Examples of such nuclei are indicated by asterisks. Scale bar represents 10 μm. Separate images of the nuclei in the boxed area are shown below, and the wild type and mutant chromosome are indicated with arrows for one nucleus. Scale bar represents 5 μm. Right: Quantification of type 1 and type 2 nuclei in two E15 ROSI-derived Xp*ΔXist* female placentas. Cells with a single green/red RNA FISH cloud and a separate red DNA FISH pinpoint were never observed.

The unexpected survival of Xp*ΔXist* female embryos might be explained by maintenance of the PSCR state of the round spermatid-derived Xp, as we initially hypothesized. However, it cannot be excluded that survival of the embryos might be explained by inactivation of the wild type Xm, replacing iXCI of the mutant Xp. In order to distinguish between these different possibilities, we analyzed *Xist* expression levels by qPCR in three E15 control male and female placentas and in the ROSI-derived Xp*ΔXist* female placentas for which RNA samples were available. As expected, male placentas showed very low *Xist* expression, which most probably reflects expression from a very small number of maternal cells through decidua contamination (Fig 3A). Conversely, *Xist* expression was very high in wild type female placentas, in accordance with maintenance of stable iXCI of the Xp in this tissue, as is required for proper extraembyonic tissue development. Surprisingly, *Xist* RNA levels of ROSI-derived Xp*ΔXist* female placentas were comparable to those of wild type female placentas (Fig 3A). Since the paternal *Xist* allele was deleted, this expression is expected to be explained by robust transcription occurring from the wild type Xm. To investigate this further, we first checked *Xist* expression by RNA FISH on E15 placenta sections obtained from ROSI-derived Xp*ΔXist* female embryos (Fig 3B). By using the Reichert’s membrane as reference for the embryonic side of the placenta, we verified that *Xist* RNA clouds were formed in the whole population of labyrinth cells of embryonic origin. Next, we performed a combined DNA/RNA FISH experiment, using two different probes; one recognizing both the wild type and mutant X chromosome, and the other recognizing only the wild type X chromosome. The results show that most cells display an *Xist* RNA cloud signal with both probes on the maternal wild type X, and that *Xist* RNA clouds are never observed on the paternal *ΔXist* X chromosome (Fig 3C). Together, these results indicate that a switch from Xp inactivation to Xm inactivation has occurred in the Xp*ΔXist* female embryos that were obtained by ROSI. This is consistent with previous data showing that *Xist* mRNA is retained by the Xi of its origin [32].

**Fig 4:**
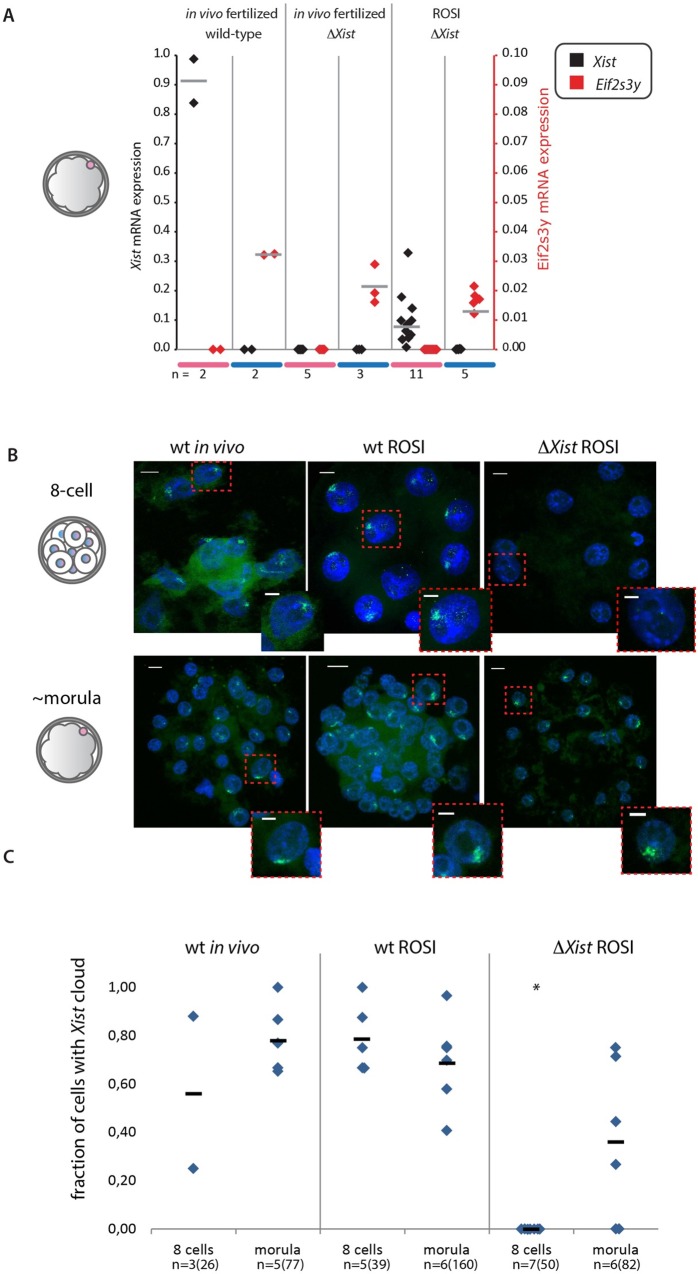
Maternal *Xist* expression in ROSI-derived Xp*ΔXist* females is variable and delayed compared to paternal *Xist* expression during iXCI. A) Dot plot showing *Xist* (black) and *Eif2s3y* (red) mRNA expression levels for RNA isolated from individual *in vivo* fertilized wt and Xp*ΔXist* male (blue) and female (pink) mouse morulas, and from ROSI-derived Xp*ΔXist* male and female morulas. Expression levels were normalized to *Actin*. Grey lines indicate the average values, n values are indicated below the graph. B) Representative images of *Xist* clouds in wild type *in vivo* fertilized female embryos, and wild type and Xp*ΔXist* ROSI-derived female embryos, analysed at the 8-cell and morula stages. Scale bars in whole embryo and single cell images represent 10, and 5 μm, respectively. C) Quantification of *Xist* cloud formation in the embryo types described in B. Individual measurements are indicated, horizontal bars represent the average value. Asterisk indicates significantly different from the corresponding stage in wild type ROSI female embryos (P = 0,00025). Numbers of analysed embryos are indicated at the bottom, and followed by total numbers of nuclei that could be scored (some nuclei were lost during procedures, and some embryos that were in the “8-cell” group contained 9 or 10 cells) in parenthesis.

Next, *Xist* mRNA expression was quantified through qRT-PCR on RNA isolated from morulas from normal fertilization with wild type and Xp*ΔXist* males, and from morulas generated by ROSI with Xp*ΔXist* round spermatids. We determined the sex of the embryos from the presence or absence of the Y-specific transcript *Eif2s3y*. As expected, *Xist* was present at very high levels in wild type female morulas, but absent from males. Also, none of the *in vivo* fertilized Xp*ΔXist* male and female morulas showed any *Xist* expression above background (Fig 4A). Interestingly, *Xist* levels were variable in ROSI-derived Xp*ΔXist* female morulas, and only one out of 11 analysed female embryos did not display any *Xist* expression. None of the wild type male morulas arising from these ROSI experiments with Xp*ΔXist* round spermatids showed *Xist* expression above background (Fig 4A). We further analysed the onset of Xm inactivation, and its variability. In wild type ROSI-derived female embryos, *Xist* clouds were prominent from the 4-cell stage onwards (Figs 1D, 1E, 4B and 4C). This pattern was not significantly different from what was observed upon *in vivo* fertilization of wild type embryos. In contrast, none of the Xp*ΔXist*, 8-cell ROSI embryos that we analysed displayed *Xist* clouds in any of the cells (Fig 4B and 4C), but we did observe clear *Xist* RNA FISH clouds at the morula stage. The number of cells with an *Xist* cloud appeared somewhat more variable compared to what was observed in wild type female ROSI derived or *in vivo* fertilized morulas, and the average fraction of positive cells was lower, when compared to *in vivo* fertilized morulas, and on the border of significance for wild type ROSI derived females (Fig 4C). Thus, Xm *Xist* activation in the Xp*ΔXist* female preimplantation embryos is delayed compared to what is observed following ROSI or *in vivo* fertilization using wild type spermatids or sperm, respectively. The observed variation in the number of cells that have formed an *Xist* cloud at the morula stage, including two embryos with no *Xist* clouds (Fig 4C), is consistent with the variation in *Xist* level that we detected in the qRT-PCR experiments (Fig 4A). The lack of clouds in some embryos is also in accordance with the notion that we did not rescue all Xp*ΔXist* females by performing ROSI.

Previous reports have shown that also in diploid parthenogenetic embryos one of the two maternal X chromosomes starts to express *Xist* around the morula stage [33,34]. It was then suggested that this could occur because a repressive imprint on the Xm *Xist* allele, preventing its expression, is not retained throughout pre-implantation development. However, in a similar situation, when *in vivo* fertilized blastocysts disomic for Xm were analysed, Xm derived *Xist* clouds were hardly ever observed [35,36]. Thus, in this latter situation, the presence of a paternal genome most likely somehow helps to maintain the maternal imprint up to the blastocyst stage. Consistent with these findings, we also did not observe *Xist* expression above background levels in any of the Xp*ΔXist* female morulas obtained by natural mating (Fig 4A).

**Fig 5:**
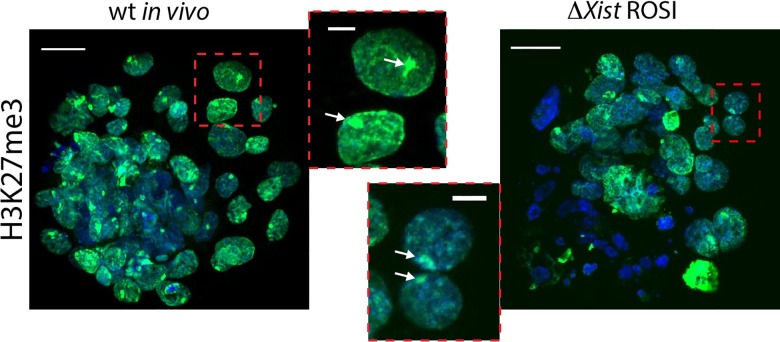
H3K27me3 marks the inactive Xm in ROSI-derived XpΔXist female blastocysts. H3K27me3 (green) marks the inactive Xp in wild type *in vivo* fertilized female blastocyst and the inactive Xm in ROSI-derived Xp*ΔXist* female blastocysts. Dapi is shown in blue. Size bars in whole embryo and single cell images represent 20, and 5 μm, respectively. H3K27me3 domain is indicated by arrows in the enlargements.

To further substantiate that the observed *Xist* clouds in the Xp*ΔXist* female morulas result in robust XCI, we investigated the immunolocalisation of H3K27me3 in female blastocysts derived from *in vivo* wild type fertilization in comparison to ROSI-derived Xp*ΔXist* female blastocysts. H3K27me3 is one of the earliest known histone modification that accompanies XCI, and is detectable as a domain covering the inactive Xp in wild type trophoblast cells [37] and Fig 5A. We also observed such H3K27me3 domains in ROSI-derived Xp*ΔXist* female embryos, (Fig 5B). These data are consistent with the occurrence of robust maternal XCI in trophoblast cells in ROSI-derived, Xp*ΔXist* female embryos. Still, not all cells may be able to activate *Xist* expression from Xm, and only if the fraction of cells that manage to do so is high enough, the embryo may be rescued.

**Fig 6:**
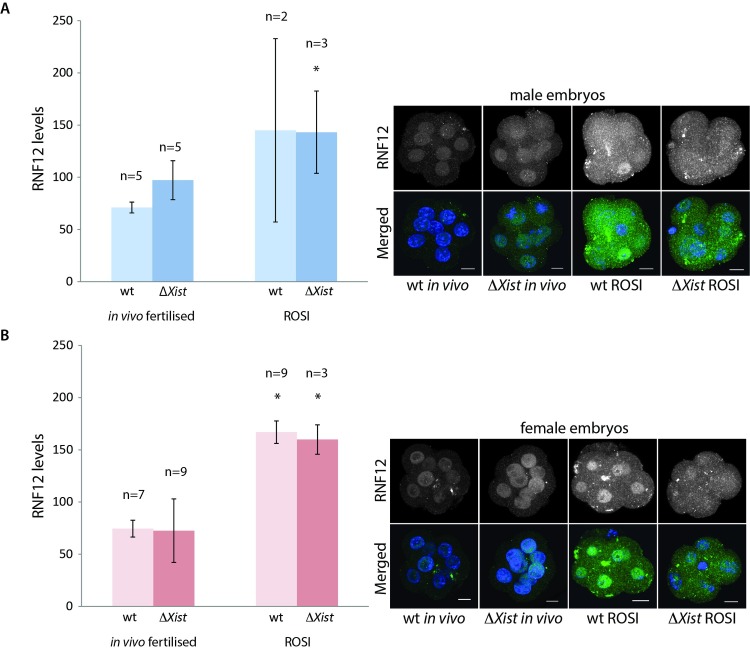
Enhanced expression of RNF12 in ROSI-derived compared to *in vivo* fertilized 8-cell embryos. A) Quantification of RNF12 protein levels ± s.d. per male 8-cell embryo as measured from immunocytochemical staining using Image J (see Materials and Methods for details). Representative images of each analysed condition are shown on the right. Size bars represent 10 μm. Asterisk indicates significant difference (P = 0,05) with *in vivo* wild type derived embryos. B) As in A, for female embryos. Asterisk indicates significant difference (P≤0,05) with *in vivo* fertilized embryos. The following P values were obtained per comparison: wt *in vivo* versus wt ROSI derived embryos P = 0,016, wt *in vivo* versus Xp*ΔXist* ROSI derived embryos P = 0,0005, *in vivo* Xp*ΔXist* versus wt ROSI derived embryos P = 0,0098 and *in vivo* Xp*ΔXist* versus Xp*ΔXist* ROSI derived embryos P = 0,004.

Recently, it was shown that expression of the X-linked XCI activator RNF12 is reduced in *in vitro* fertilized mouse embryos, and that this causes impaired iXCI, leading to skewed sex ratios of the offspring [38]. In our ROSI model, we anticipated an opposite situation with high *Rnf12* expression, since *Rnf12* is one of the X-linked genes that becomes specifically reactivated in spermatids [5]. If this status is maintained upon ROSI, then it may lead to higher RNF12 levels compared to what is observed following fertilization with mature sperm. We analysed RNF12 protein levels at the eight-cell stage, in wild type and Xp*ΔXist* ROSI derived, and *in vivo* fertilized female and male embryos. This time point was chosen because it is just prior to the initiation of *Xist* cloud formation in the Xp*ΔXist* female ROSI embryos. Interestingly, the overall RNF12 level was increased approximately three-fold in all ROSI-derived embryos compared to *in vivo* fertilized embryos (Fig 6A and 6B). However, no difference between male and female embryos was noted. In addition, RNF12 levels of all *in vivo* derived embryos, fertilized either by wild type or *ΔXist* sperm, were similar (Fig 6A and 6B). This latter observation indicates that failure to inactivate the paternal X does not lead to a measurable significant increase in RNF12 levels using this type of semi-quantitative immunocytochemical analysis.

RNF12 expression from the paternally inherited postmeiotically reactivated X chromosome by itself cannot easily explain our findings, since ROSI-derived males also display high RNF12 levels. Somehow, the injection of a round spermatid nucleus must either transfer a substantial amount of very stable RNF12 protein or mRNA, or other, autosomal spermatid-expressed genes ensure continuous *Rnf12* expression from the maternal X in males, and perhaps from both X chromosomes in females. It might be suggested that the observed enhanced RNF12 expression could contribute to the ability of Xp*ΔXist* embryos to overcome the maternal imprint on the Xic, and allow maternal XCI. However, other X-linked factors are most likely more critically involved in lowering the threshold for activation of the Xm *Xist* gene in the Xp*ΔXist* female embryos, because Xm inactivation was never observed in male ROSI embryos. Alternatively, or in addition, the chromatin structure of the paternal X chromosome, being heavily marked by silencing histone modifications, may titrate away factors that are important for maintenance of the inactive status of the maternal *Xist* gene.

In future experiments, comparative global gene expression analyses of ROSI derived and ICSI derived embryos might be used to identify novel XCI factors involved in both imprinted and random X chromosome inactivation. From this perspective, it will also be interesting to compare the gene expression profiles of purified round spermatids from C57BL/6 mice and CAST/EiJ mice, since we failed to rescue the lethality of paternal *Xist* deletion using ROSI on the latter genetic background. Microarray analyses of gene expression using total testis mRNAs of *M*. *musculus musculus* and *M*. *musculus castaneus* identified a relatively small number of differentially expressed spermatogenesis genes [39]. In this dataset, expression of *Rnf12* was not significantly different between the *M*. *musculus* subspecies [39]. Thus, we speculate that differences in regulation of expression of genes other than *Rnf12* may be critical for inducing maternal *Xist* expression in ROSI derived Xp*ΔXist* embryos on the C57BL/6 background only.

**Fig 7:**
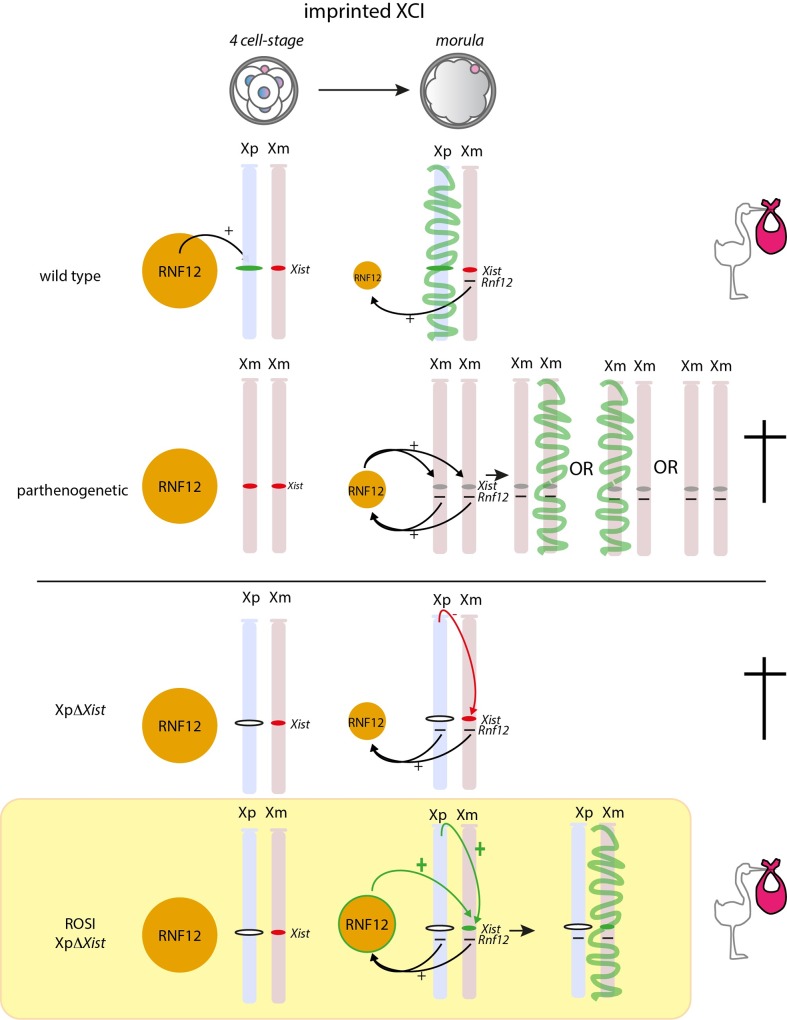
Schematic representation of critical factors during iXCI in wild type and parthenogenetic females, compared to Xp*ΔXist* females arising from in vivo fertilization or ROSI. Xp (blue) and Xm (pink) are schematically drawn, and the *Xist a*nd *Rnf12* loci are indicated where relevant. RNF12 protein levels are represented by the size of the orange circle. A green *Xist* locus indicates that it is primed for expression, whereas a red locus indicates that it is repressed. An open Xist locus represents the *ΔXist* allele. During normal iXCI, that initiates around the four cell-stage, high maternally regulated RNF12 levels ensure *Xist* expression from Xp, establishing iXCI by the morula stage (green signal on Xp representing the *Xist* cloud). In parthenogenic embryos, the imprint on Xm is lost by the morula stage and *Xist* can be activated in a manner that appears to be regulated by dosage dependent XCI activators such as RNF12 (effect symbolized by two black arrows coming from the *Rnf12* loci), but this is inefficient. In contrast, when *Xist* is deleted from Xp, iXCI cannot be induced, most likely because the presence of a paternal genome helps to maintain repression of the maternal *Xist* gene (red arrow). Upon ROSI, round spermatid-specific epigenetic regulation (green arrow), possibly in combination with the double dosage of X chromosomes, allows activation of Xm by the morula stage. In addition, RNF12 levels are relatively high and may also aid in this process.

In the model in Fig 7, we schematically depict the differences between regulation of iXCI following *in vivo* fertilization, ROSI, or induction of parthenogenesis. When iXCI is initiated in wild type embryos carrying an Xp and an Xm (as opposed to two Xms in the parthenogenic situation), the Xp most likely is more responsive to XCI *trans* activator(s) such as RNF12 than the Xm. This differential response is related to an imprint of the *Xist* promoter on the Xm, which prevents *Xist* expression, and which is absent from the promoter on Xp [19]. In addition, Xp may carry an (MSCI-dependent) imprint to facilitate *Xist* expression. At this stage, RNF12 expression is relatively high, due to the maternally provided store, and paternal *Xist* activation occurs independent of the X:A ratio. Transcription of the XCI activator(s) would reach the threshold for *Xist* expression from the Xp in all blastomeres by the 4-cell stage, but virtually never reach the threshold for activation of *Xist* expression from Xm. When the paternal copy of *Xist* is deleted, iXCI can not occur, and the maternal *Xist* gene remains repressed due to a paternal inhibitory effect that is missing in parthenogenetic embryos. ROSI somehow leads to elevated levels of RNF12 in morulas, but this by itself will not be enough to activate Xm in ROSI-derived males, consistent with earlier findings using *Rnf12* overexpression [38]. Somehow, either the presence of two X chromosomes, or the specific epigenetic constitution of the Xp, contributes to efficient stimulation of *Xist* expression from Xm. Subsequent Xm silencing most likely allows rescue of Xp*ΔXist* females. In addition, prior to the establishment of *Xist* mediated Xm inactivation, the silencing epigenetic marks that are carried by the round spermatid-derived Xp may also contribute to a more optimal gene-expression balance. This may exert an additional positive effect on the fitness of the ROSI-derived Xp*ΔXist* female embryos.

Taken together, the present experiments have demonstrated that ROSI allows activation of *Xist* transcription from the Xm in preimplantation mouse embryos in the absence of a paternal *Xist* gene. We propose that correct regulation of expression of X-linked *trans* activators of XCI from both the paternal and maternal X chromosome is of critical importance in iXCI in mouse.

In humans, X chromosome inactivation is initiated later than in mouse, and most likely is not imprinted (reviewed in [40]). Still, our results do provide evidence that disturbances of the paternal epigenome impact on embryonic gene regulation, and this is relevant for considerations on human embryo quality. In humans, there is an increase in the histone:protamine ratio when sperm from male factor subfertility patients is compared with sperm from fertile men [41,42]. Also, when sperm is extracted from the testis and used for ICSI, it cannot be excluded that spermatids with an incomplete histone-to-protamine transition are selected for injection into oocytes, so that ICSI resembles ROSI. Furthermore, the birth of 14 ROSI-derived babies was recently described [43], making careful assessment of possible associated epigenetic risks more topical than ever.

Future clinical and basic animal research should go hand in hand to evaluate if there is a relation between embryo paternal epigenome quality and oocyte injection using spermatids or spermatozoa in which the histone-to-protamine transition has not been completed or is disturbed.

## Materials and Methods

### Ethics statement

For all experiments we aimed to reduce pain and stress as much as possible by housing animals in groups whenever possible, and using appropriate anesthetic agents during operation, followed by treatments to reduce pain. Animals more than one week old were killed using cervical dislocation. Embryos collected after day 13 of embryonic development, and pups younger than 1 week old were killed by decapitation and immediate collection of heads in liquid nitrogen. All animal experiments were approved by and were performed in strict accordance with the recommendations by the local animal experiments committee DEC-consult (approval numbers EMC2448 and EMC3200).

### Animals

B6D2F1 mice (C57BL/6 × DBA/2) were used as oocyte donors. We used B10CBA females that were mated with vasectomized males as pseudopregnant surrogates for transfer of ICSI- and ROSI-derived two-cell stage embryos. C57Bl6 mice carrying an *Xist* deletion (*ΔXist*) were those originally generated by Csankovszki and colleagues [29], the allele was also crossed into a CAST/EiJ background for several generations, but round spermatids isolated form *ΔXist* males with this background did not result in retrieval of viable female embryos. Control wild type C56BL/6 males were also used as spermatid and spermatozoa donors. To obtain embryos from *in vivo* fertilized oocytes, superovulated B6D2F1 females (see below for the superovulation protocol) were mated with wild type or *ΔXist* males and zygotes were retrieved from the oviduct and cultured for different applications as described in the expanded view.

### Microinsemination with round spermatids (ROSI)

ROSI was carried out as described previously [44] with minor modifications:

#### ROSI: Oocyte collection

Mature oocytes were collected from the oviducts of 6- to 16-wk-old B6D2F1 female mice (Harlan) that had been induced to superovulate with 5 IU pregnant mare’s serum gonadrotopin (PMSG; Intervet), followed by 5 IU human chorionic gonadotropin (hCG; Intervet) 48 h later. Oocytes were collected from oviducts approximately 16 h after hCG injection and treated with 80 IU ml^-1^ hyaluronidase (Sigma) until the cumulus cells dispersed. The oocytes were then placed in G-1 PLUS medium (Vitrolife), covered with mineral oil (Sigma), and stored at 37°C (5% CO2:95% air). Before injection, oocytes were placed into Ca^2+^-free M16 containing 10 mM SrCl_2_ (Sigma) for 60 min. Oocytes were injected in MEMα medium (Life Technologies) supplemented per 500 ml with 2.5 g HEPES, 684 mg 50% sodium lactate solution, 55 mg sodium pyruvate, 70 mg L-glutamin, 6% (v/v) fetal calf serum (pH set at 7.2).

#### ROSI: Spermatogenic cell suspension preparation

To collect round spermatids, seminiferous tubules of the testes from male mice were gently minced using two blunt ended curved forceps, and single cells were suspended in G-MOPS PLUS medium (Vitrolife).

#### ROSI: Microinsemination with round spermatids

ROSI was carried out at room temperature. The cover of a plastic dish was used as a microinjection chamber. A row of three 10 μl drops containing HEPES-buffered MEMα + supplements (for oocytes), 12% polyvinylpyrrolidone (PVP; Irvine scientific) in G-MOPS PLUS, and of the spermatogenic cell suspension, was placed on the bottom of the dish and covered with mineral oil. The dish was placed on the stage of an inverted microscope. The nuclei of the round spermatids were collected from the spermatogenic cell suspension drop by gentle pipetting using a 5 μm Piezo Drill Micropipette (Humangen) until the nuclei of the round spermatids had lost all cytoplasm and could then be collected, transferred to the clean PVP drop and subsequently used for microinjection. An oocyte was held to the holding pipette with the metaphase II spindle at either the 12 or the 6 o'clock position. The zona pellucida was breached by a laser applied pulse (XY clone, Hamilton Thorne) and the plasma membrane was subsequently penetrated by using a piezo-activated device (Burleigh). The spermatid nucleus was readily expelled into the ooplasm.

Injected oocytes were then transferred to G-1 PLUS medium and cultured for 24 and 96 h, to examine their development *in vitro*.

### Microinsemination with mature spermatozoa (ICSI)

ICSI was carried out as described previously [45].

### Embryo culture and transfer

Injected oocytes were cultured for 24–30 h in G-1 PLUS medium until the two-cell stage. Thereafter, 10–15 two-cell embryos were transferred to each oviduct of surrogate females on day 1 of pseudopregnancy. Alternatively, embryos were cultured up to the 4-cell, 8-cell, or morula stage in G-1 PLUS medium and further processed for different applications as described below.

### Chromosome spread preparations

Zygotes or two-cell embryos were incubated with colcemid (1.5 μg/ml) to arrest cells at prometaphase until pronuclei had disappeared. To obtain chromosome spreads, after zona pellucida removal with Acidic Tyrode’s Solution (Sigma), arrested zygotes were incubated in hyposolution (25% v/v FCS, 0.5% w/v sodium citrate) for 5 min and subsequently transferred to a drop of fixative (1% v/v paraformaldehyde, 0.2% v/v Triton X-100, 0.1 mM dithiothreitol, pH 9.2) on a glass slide. After horizontal drying for 1 h, the slides were washed with 0.08% Photo-Flo (Kodak) and air dried. All slides were stored at −20°C until further use.

### Decondensation of mouse caput sperm

Decondensation of wild type mouse caput sperm was performed as described previously [24].

### Preparation of spread spermatid nuclei

Nuclei of wild type mouse spermatogenic cells were spread as previously described [46].

### Immunofluorescence

For immunofluorescence stainings, the zona pellucida of the 8-cell embryos was removed with incubation in Acidic Tyrode’s Solution (Sigma) at room temperature for 1–2 min. Afterwards, embryos were washed in M2 medium, fixed in 4% PFA for 15’ at room temperature and then washed again in M2 medium. Subsequently, embryos and slides containing zygote or embryo chromosome spreads, decondensed sperm, or spread spermatid nuclei, were rinsed in PBS-phosphate-buffered saline PBS-T (PBS, 0.01% v/v Tween-20) and blocked with blocking solution (PBS-T, 2% w/v bovine serum albumin (BSA fraction V), 5% v/v normal goat serum) for 30 minutes and incubated with primary antibodies at 4°C overnight. The following antibodies were used in this study: rabbit polyclonal against H3K9me3 (1:200, Abcam Ab8898-100), mouse monoclonal anti H3.1/2 (1:1000, gift from dr. P. de Boer, for validation see [4]), mouse monoclonal against RNF12 (1:50 Abnova), and human centromere autoantigen (ACA, 1:1000, Fitzgerald Industries, 90C-CS1058). After washing with PBS-T, slides were incubated with the appropriate secondary antibodies for 1 hour, washed with PBS-T and mounted with ProLong Gold mounting solution for DNA counterstaining. Images were obtained using a LSM700 confocal laser scanning microscope (Zeiss) and processed with Fiji and Adobe Photoshop CS3 software. Imaging of RNF12 stained embryos was performed using the same exposure time for each embryo. Quantification of total RNF12 levels per embryo was performed using Image J (Fiji) software. Subsequently, statistical significance was determined by Student’s t test (*P≤0.05, significant).

### RNA/DNA FISH on preimplantation embryos

Pre-implantation embryos were treated with Acidic Tyrode’s Solution (Sigma) to remove the zona pellucida. The method for *Xist* RNA-FISH has been described [32,47], and we used a 5.5 Kb BglII cDNA fragment,covering exons 3-7(in part) as a probe. For DNA FISH on chromosome spreads of prometaphase arrested embryos and on 8 cell embryos after RNA FISH or immunostaining (for sex determination), slides were denatured in 70% v/v formamide/2x SSC/10mM phosphate buffer for 5’ at 78°C followed by dehydration in ice cold ethanol series (70%, 85% and 100%) 3 minutes each. Slides were left to dry for a few minutes at room temperature and then, the same *Xist* probe used for RNA FISH was applied on the slide. Detection was performed as for RNA FISH.

### Placenta cryosections

Placentas were removed at E15. The tissues were snap frozen and stored at -80°C until use. For RNA FISH, 14 μm-thick frozen sections were made from frozen tissues on a cryostat and mounted on glass slides. Sections were briefly air-dried, extracted with 0.5% Triton X-100 in phosphate-buffered saline (PBS) on ice, fixed in 4% formaldehyde, 5% acetic acid for 18 min at room temperature, washed 3 times in PBS for 5 min each, dehydrated in 70–100% ethanol series and air-dried. Then the probes were applied. For DNA FISH the same procedure as described above for RNA/DNA FISH on preimplantation embryos’ was followed. Both for RNA and DNA FISH, an additional *Xist* probe was used that is specific for the wild type X chromosome. This probe covers a 8.4 Kb fragment lying within the deleted area of the Δ*Xist*. It was generated by combination of PCR products obtained with the following primer sets: Fwprom CCCTCTGGAAGAGCAGTCAG and Rvprom GCCATAAGGCTTGGTGGTAG (~1,7Kb), Fw1 GCCAACCAATGAGACCACTT and Rv1 TGGCATGATGGAATTGAGAA(~2.5Kb), Fw2 CTACCCACCCCAGTACATGC and Rv2 TTGGCTCAGTGCTTATGGTG (~2.1Kb), Fw3 CAGTTGCCTTCTCCTTGCTC and Rv3 AGCTGTTAGTGCCGTCCAGT (~2.1Kb). The PCR conditions were: initial denaturation 94°C for 5 min, followed by 35 cycles of 94°C 30 sec, 55°C 30 sec, 72°C 3 min, and final extension at 72°C for 5 min. PCR products were loaded on a 1% agarose gel, bands were extracted and DNA was isolated using NucleoSpin Extract II (Macherey Nagel) according to the manufacturer’s protocol. Subsequently, the probe was made using the Biotin Nick translation mix (Roche diagnostics), according to the manufacturer’s instructions.

### Genotyping PCR

The primer pairs used to assess the genotype of the mice for the presence or absence of the *Xist* deletion, and to detect *Ube1x* and *Ube1y* have been previously described (*Xist* deletion: [48], *Ube1x/y*: [49]). For *Sry* we used forward primer 5’GTGGTCCCGTGGTGAGAG3’, and reversed primer 5’TTTTGTTGAGGCAACTGCAG3’, generating a 250bp fragment. PCR conditions were as follows: Initial hold for 2 minutes at 98°C, followed by 35 cycles of 98°C for 10 seconds, 63°C for 15 seconds, and 72°C for 30 seconds, and finally 72°C for 5 minutes.

### Quantitative PCR analyses

For quantitative RT–PCR (RT–qPCR) of single embryos, the Taqman Cells-to-Ct Kit (Applied Biosystems) was used according to the manufacturer's protocol. All samples were analyzed in triplicate in a 10 μl final reaction volume using the BioRad CFX 384 Real-time System. The reaction mixture contained SYBR Green PCR Master Mix (Applied Biosystems), primers (for *Actin*, *Xist*, or *Eif2s3y*) and 2.5 μl of cDNA. The following primers were used: *Xist* for GGATCCTGCTTGAACTACTGC and *Xist* rev CAGGCAATCCTTCTTCTTGAG [50], *Actin* for AACCCTAAGGCCAACCGTGAAAAG and rev CATGGCTGGGGTGTTGAAGGTCTC, *Eif2s3y* for CCAGGGACCAAAGGAAACTT and rev TAGCCTGGCTTTCTTTCACC [51].

For copy number qPCR on genomic DNA, primers were designed for the X chromosome on the *Tsix* promoter region (for CCGAGATATCCACGCATCTT and rev AGCTGGCTATCACGCTCTTC) and for chromosome 12 on the *Rex1* allele (for GGTGCAAGAAGAAGCTGAGG and rev GTTTCGAGCTCTCCGTGAAG).

After an initial hold at 94°C for 2 minutes, reaction mixtures underwent 40 cycles of 30s at 94°C, 30s at 60°C, and 30s at 72°C. Results were expressed as Cycle threshold (Ct) values. Gene expression levels were normalized over *Actin* gene expression, according to the 2^- ΔCT^ method [52]. In order to be able to use a relative quantification approach to compare expression levels we ensured that the primer pairs have similar amplification efficiencies (E = 100 ± 10%).

## Supporting Information

S1 Fig Genotyping of ROSI and ICSI embryos and pups. A) DNA was isolated from E15 ROSI embryos and genotyped using primer sets amplifying either *Ube1x* and *Ube1y* or *Sry* (top). In addition, PCR was performed to detect the presence of *Xist* loci (wild type (wt) and knockout (ko)). All PCRs from XX female embryos should display a band for both alleles. One female was found to carry only a single X chromosome (20, X; XO). Results are grouped per experiment (1–4) in chronological order. In experiment 4, DNA was isolated for only 1 of the 5 males that were obtained. For the other four embryos, sex assignment was based solely on the presence of testes, since the results were congruent with the morphological assessment in all previous experiments. B) DNA was isolated from E15 ICSI embryos and genotyped using primer sets amplifying *Ube1x* and *Ube1y*. All embryos were male, a control female (CF) is shown for comparison. Results are grouped per experiment (1–4) in chronological order. C) DNA was isolated from P1 ROSI embryos and genotyped using primer sets amplifying *Ube1x* and *Ube1y* (CF; control female, CM; control male). Results are grouped per experiment (1–2) in chronological order.
(TIF)

S1 Table Sex ratios reported in published mouse ROSI experiments. #: number; m: male; f: female
(PDF)
