# Supplementary figures and images for: Round Spermatid Injection Rescues Female Lethality of a Paternally Inherited Xist Deletion in Mouse
Source: PLoS Genet. 2016 Oct 7;12(10):e1006358. doi: 10.1371/journal.pgen.1006358 (PMC5065126; doi:10.1371/journal.pgen.1006358)

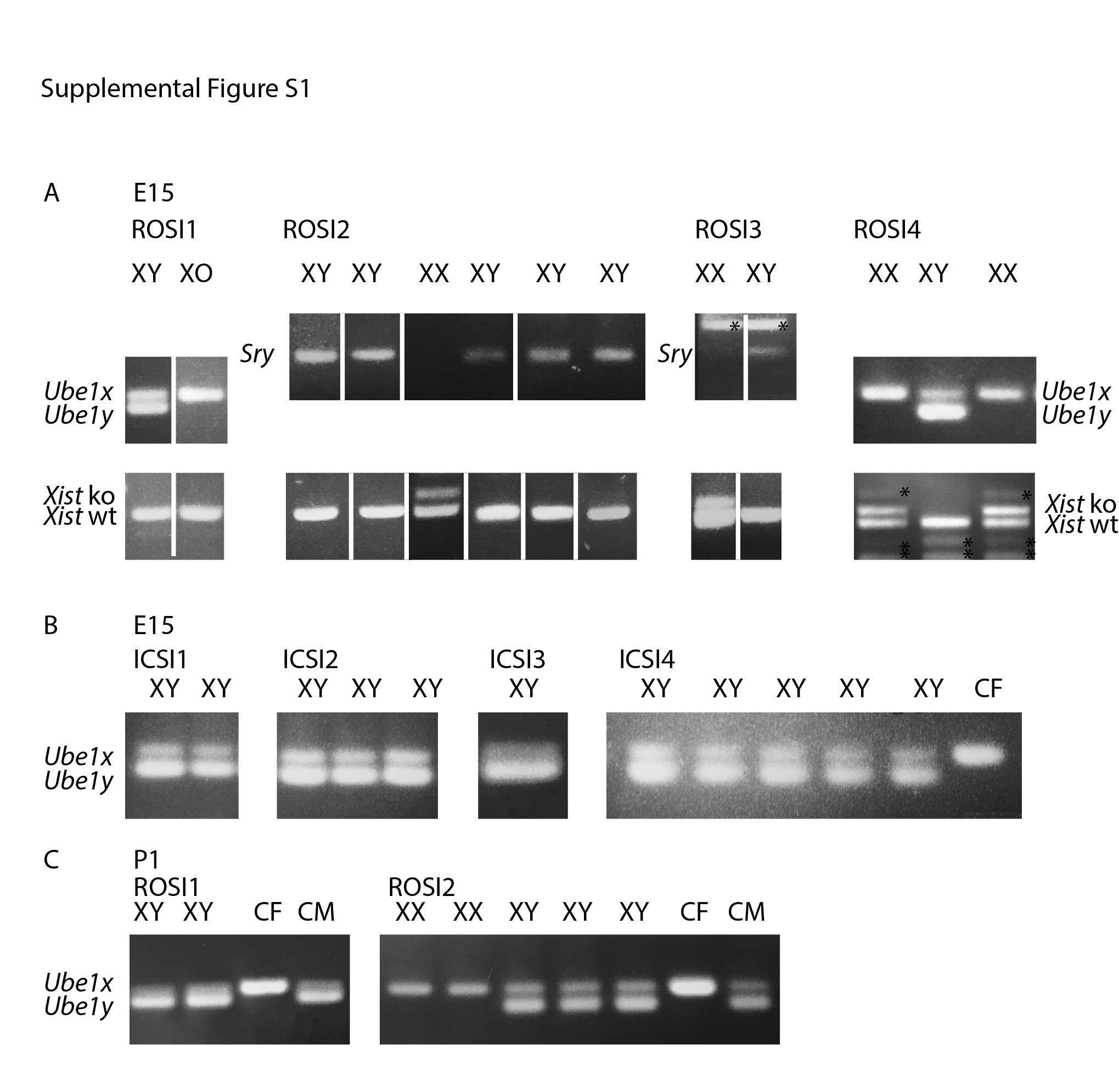

Supplement: S1 Fig — A) DNA was isolated from E15 ROSI embryos and genotyped using primer sets amplifying either Ube1x and Ube1y or Sry (top). In addition, PCR was performed to detect the presence of Xist loci (wild type (wt) and knockout (ko)). All PCRs from XX female embryos should display a band for both alleles. One female was found to carry only a single X chromosome (20, X; XO). Results are grouped per experiment (1–4) in chronological order. In experiment 4, DNA was isolated for only 1 of the 5 males that were obtained. For the other four embryos, sex assignment was based solely on the presence of testes, since the results were congruent with the morphological assessment in all previous experiments. B) DNA was isolated from E15 ICSI embryos and genotyped using primer sets amplifying Ube1x and Ube1y. All embryos were male, a control female (CF) is shown for comparison. Results are grouped per experiment (1–4) in chronological order. C) DNA was isolated from P1 ROSI embryos and genotyped using primer sets amplifying Ube1x and Ube1y (CF; control female, CM; control male). Results are grouped per experiment (1–2) in chronological order. (TIF) [file pgen.1006358.s001.tif]
