# Supplementary material for: Round Spermatid Injection Rescues Female Lethality of a Paternally Inherited Xist Deletion in Mouse
Source: PLoS Genet. 2016 Oct 7;12(10):e1006358. doi: 10.1371/journal.pgen.1006358 (PMC5065126; doi:10.1371/journal.pgen.1006358)
Supplement: S1 Table — #: number; m: male; f: female (PDF) [file pgen.1006358.s002.pdf]

Supplemental Table S1: Sex ratios reported in published mouse ROSI experiments

Author	system	# embryos	# pups	m	f	m /f	% survival	sex ratio
Kimura et al., 1995 [1]	wt ROSI	131	37	16	19	2	28,24%	0.84
Ogura et al., 1996 [2]	ICGN males ROSI	53	9	4	5		16,98%	0.80
Sasagawa and Yanagimachi. 1997 [3]	wt ROSI	46	11	4	7		23,91%	0.57
Sasagawa and Yanagimachi. 1997 [3]	Cryptorchid male ROSI	40	9	5	4		22,50%	1.25
Sasagawa and Yanagimachi. 1997 [3]	reversal Cryptorchid males 6w	41	9	4	5		21,95%	0.80
Sasagawa and Yanagimachi. 1997 [3]	reversal Cryptorchid males 14w	43	10	7	3		23,26%	2.33
Sasagawa et al., 1998 [6]	ROSI with spermatids from immature mice	148	29	13	16		19,59%	0.81
Sasagawa et al., 1998 [6]	wt ROSI	46	11	5	6		23,91%	0.83
Sasagawa et al., 1998b [7]	ROSI with Balb/c (hybrid sterile)	55	12	7	5		21,82%	1.40
Sasagawa et al., 1998b [7]	ROSI with B6D2F1 (hybrid fertile)	61	14	6	8		22,95%	0.75
Marh et al., 2003 [10]	wt ROSI	60	16	9	7		26,67%	1.29
Yanagimachi et al., 2004 [11]	ROSI wit *qk/qk* spermatids	96	18	8	10		18,75%	0.80
TOTALS		820	185	88	95	2	22,56%	0.93

References

1. Kimura Y, Yanagimachi R (1995) Mouse oocytes injected with testicular spermatozoa or round spermatids can develop into normal offspring. Development 121: 2397-2405.
2. Ogura A, Yamamoto Y, Suzuki O, Takano K, Wakayama T, et al. (1996) In vitro fertilization and microinsemination with round spermatids for propagation of nephrotic genes in mice. Theriogenology 45: 1141-1149.
3. Sasagawa I, Yanagimachi R (1997) Spermatids from mice after cryptorchid and reversal operations can initiate normal embryo development. JAndrol 18: 203-209.
4. Sasagawa I, Tomaru M, Adachi Y, Kubota Y, Nakada T (1997) Simultaneous injection of round spermatid nuclei from mice and hamster oscillogen can initiate the normal development of mouse embryos. J Urol 158: 2006-2008.
5. Suzuki K, Yanagida K, Yanagimachi R (1998) Comparison of the media for isolation and storage of round spermatid nuclei before intracytoplasmic injection. J Assist Reprod Genet 15: 154-157.
6. Sasagawa I, Tateno T, Adachi Y, Kubota Y, Nakada T (1998) Round spermatids from prepubertal mouse testis can develop into normal offspring. J Androl 19: 196-200.
7. Sasagawa I, Tateno T, Yazawa H, Ichiyanagi O, Ishigooka M, et al. (1998) Round spermatids from hybrid sterile mice can initiate normal embryo development. Hum Reprod 13: 3099-3102.
8. Sakurai A, Oda S, Kuwabara Y, Miyazaki S (1999) Fertilization, embryonic development, and offspring from mouse eggs injected with round spermatids combined with Ca2+ oscillation-inducing sperm factor. Mol Hum Reprod 5: 132-138.
9. Meng X, Akutsu H, Schoene K, Reifsteck C, Fox EP, et al. (2002) Transgene insertion induced dominant male sterility and rescue of male fertility using round spermatid injection. Biol Reprod 66: 726-734.
10. Marh J, Tres LL, Yamazaki Y, Yanagimachi R, Kierszenbaum AL (2003) Mouse round spermatids developed in vitro from preexisting spermatocytes can produce normal offspring by nuclear injection into in vivo-developed mature oocytes. Biol Reprod 69: 169-176.
11. Yanagimachi R, Wakayama T, Kishikawa H, Fimia GM, Monaco L, et al. (2004) Production of fertile offspring from genetically infertile male mice. Proc Natl Acad Sci U S A 101: 1691-1695.
